# Lung ultrasound score in dogs and cats: A reliability study

**DOI:** 10.1111/jvim.16956

**Published:** 2023-11-27

**Authors:** Stefano Oricco, Daniele Medico, Ilaria Tommasi, Richard Marcello Bini, Roberto Rabozzi

**Affiliations:** ^1^ Centro Veterinario Imperiese Imperia Italy; ^2^ Department of Veterinary Sciences University of Parma Parma Italy; ^3^ Policlinico Veterinario Roma Sud Roma Italy

**Keywords:** agreement, lung aeration, pattern recognition, point‐of‐care ultrasound, repeatability, respiratory distress, thoracic ultrasound

## Abstract

**Background:**

Lung ultrasound (LUS) is a noninvasive tool for examining respiratory distress patients. The lung ultrasound score (LUSS) can be used to quantify and monitor lung aeration loss with good reliability.

**Hypothesis/Objectives:**

Assess the reliability of a new LUSS among raters with different levels of experience and determine how well the same raters agree on identifying patterns of LUS abnormalities.

**Animals:**

Forty LUS examinations of dogs and cats and 320 videos were reviewed from a digital database.

**Methods:**

Retrospective reliability study with post hoc analysis. Protocolized LUS were randomly selected; intrarater and interrater reliability of the LUSS and pattern recognition agreement among 4 raters with different levels of experience in LUS were tested.

**Results:**

The intrarater intraclass correlation coefficient (ICC) single measurement, absolute agreement, and 2‐way mixed effects model was 0.967 for the high‐experience rater (H‐Exp), 0.963 and 0.952 for the medium‐experience raters (M‐Exp‐1; M‐Exp‐2), and 0.950 for the low‐experience rater (L‐Exp). The interrater ICC average measurement, absolute agreement, and 2‐way random effects model among the observers was 0.980. The Fleiss' kappa (k) values showed almost perfect agreement (k = 1) among raters in identifying pleural effusion and translobar tissue‐like pattern, strong agreement for A‐lines (k = 0.881) and B‐lines (k = 0.806), moderate agreement (k = 0.693) for subpleural loss of aeration, and weak agreement (k = 0.474) for irregularities of the pleural line.

**Conclusions and Clinical Importance:**

Our results indicate excellent intra‐ and interrater reliability for LUS scoring and pattern identification, providing a foundation for the use of the LUSS in emergency medicine and intensive care.

**Abbreviations**
A: A‐Lines
B: B‐Lines
CI: confidence interval
H‐Exp: high‐experience rater
ICC: intraclass correlation coefficient
IPL: irregular pleural line
L‐Exp: low‐experience rater
LUS: lung ultrasound
LUSS: lung ultrasound score
M‐Exp‐1: medium‐experience rater 1
M‐Exp‐2: medium‐experience rater 2
PE: pleural effusion
SubPl‐LoA: subpleural loss of aeration
TrL‐TLP: translobar tissue‐like pattern

## 1 INTRODUCTION

Lung ultrasound (LUS) is a rapid, noninvasive valuable tool for assessing respiratory distress patients. Qualitative LUS is a subjective characterization of the lung and is based on the description of artifacts, analysis of the pleura, and visualization of other LUS abnormalities that are not artifacts, such as pleural effusion or consolidations, to help determine the cause of acute respiratory distress. A quantitative approach is helpful in monitoring patients during treatment.^1^ For this purpose, several scoring systems have been developed in human medicine to measure the extent of aeration loss objectively.^2^ Various patterns can be recognized in LUS^3^:

A‐profile: a normal lung, characterized by the presence of horizontal artifacts (A‐lines) that should be associated with a dynamic sign such as lung sliding to confirm a normal lung touching the chest wall, with normal to and from movement of the visceral and parietal pleural surface.
B‐profile (interstitial syndrome): at least 3 vertical artifacts (B‐lines) in 1 quadrant and a decrease in aeration depending on how much these artifacts affect the pleural surface.^4^
Irregular pleural line: a hypoechoic band with an irregular boundary; the pleural line can be rough or patchy, even discontinuous, during loss of aeration; it is important to differentiate it from pleural thickening, which is only present in pleural diseases such as fibrosis or chronic inflammation. Pleural irregularity results from mild peripheral loss of lung aeration, and the observed increase in thickness is not to be attributed to pleural tissue. For this reason, “irregular” is more suitable.
Tissue‐like pattern (alveolar syndrome)^5^: complete loss of aeration in the affected area caused by consolidation or atelectasis. Consolidation occurs when inflamed or neoplastic tissue replaces aerated tissue, whereas atelectasis is a loss of aeration without any increase in tissue. Both can be *peripheral* or *translobar*, with the former describing subpleural or focal loss of aeration.
Pleural effusion: fluid collection in the pleural space, which could be anechoic, hypoechoic, flocculated, finely corpuscular, or anechoic with isolated fibrin branching.^6^

In small animal veterinary medicine, different B‐line scoring systems have been developed,^7^, ^8^, ^9^, ^10^, ^11^, ^12^ sometimes including the description of tissue‐like patterns.^13^, ^14^ Such scoring systems have been used to assess the severity of specific diseases, with higher sensitivity and agreement of LUS, compared to computed tomography of the chest, than chest radiographs.^14^ The scores are determined by counting B‐lines, which are ranked on an ordinal scale from 0 to 4/5; the lower ranks (1‐3) reflect the precise number of B‐lines, whereas the highest ranks indicate a large number or coalescent B‐lines that are difficult to quantify numerically. In these B‐line scoring systems, tissue‐like patterns always were quantified as the highest score, regardless of their extension.

A recent study^4^ introduced a lung ultrasound score (LUSS) for humans that calculates the percentage of the entire pleural surface occupied by the main pattern.

Our primary aim was to evaluate the reliability of this new LUSS across raters with different levels of experience. The secondary aim was to assess the level of agreement among raters to accurately classify different patterns.

## 2 MATERIALS AND METHODS

Ours was a retrospective reliability study with post hoc analysis of the ultrasonographic examinations performed by the same operator and archived in the digital database of the Centro Veterinario Imperiese; all records of dogs and cats that had LUS between January 1, 2021, and December 31, 2021 were consecutively selected from the digital archive and anonymized.

Our center follows a standardized protocol for all LUS examinations conducted. Patients are positioned in sternal recumbency or standing, with hair clipped and skin covered with ultrasound gel and alcohol; each hemithorax is divided into 4 quadrants (caudal‐dorsal, cranial‐dorsal, cranial‐ventral, and caudal‐ventral) as described previously.^15^ The sixth intercostal space marks the boundary between the cranial and caudal regions, whereas the elbow serves to divide the dorsal and ventral areas. A scan of every quadrant is performed by placing the probe in each intercostal space and aligning it parallel to the axis of the ribs (transversal scan) to explore the entire pleural surface and avoid any rib shadowing.

The inclusion criteria were that all videos had to be correctly performed by a high‐experience (H‐Exp) operator in LUS. To ensure a thorough examination, in our center, a standardized protocol is used to perform LUS, and involves scanning and recording 4 quadrants: caudal‐dorsal, cranial‐dorsal, cranial‐ventral, and caudal‐ventral in that order on the left side, followed by the same order on the right. Within each quadrant, a single cine loop is saved wherein the operator scans each intercostal space in a zig‐zag pattern; videos are recorded prospectively, stopping the recording at the end of the scan of the entire quadrant, and trying to maintain a consistent speed and avoid dwelling on pathological points that could alter post hoc evaluation. All of the examinations are recorded for the purpose of being reviewed, and LUSS is recalculated post hoc. To avoid any misinterpretation during the study, examinations that were not accurately recorded, either because of short videos with rapid scans, loss of contact between the probe and the skin, or missing videos, were excluded.

All of the examinations were performed using a linear ultrasound transducer L12‐4s (3‐13 MHz bandwidth) and an M9 ultrasound machine (Mindray, Nanshan, Shenzhen, PR China).

### 2.1 Intrarater and interrater reliability of the LUSS

Intrarater and interrater reliability were tested on 4 raters with different levels of experience in LUS. The H‐Exp rater was compared to 2 medium‐experience raters (M‐Exp‐1; M‐Exp‐2) and 1 low‐experience rater (L‐Exp) in LUS. The experience level was defined as the number of ultrasound examinations performed (<50 examinations for L‐Exp, 50 to 100 examinations for M‐Exp‐1 and M‐Exp‐2, and >500 examinations for H‐Exp).

The less experienced and moderately experienced raters received a 3‐hour training session from the more experienced rater, focusing on pattern definition and LUSS assignment method.

To determine the appropriate sample size for our study, we conducted an a priori power analysis using a web‐based sample size calculator (https://wnarifin.github.io/ssc_web.html).^16^, ^17^ We used published data from the human medical literature^4^ to obtain the expected intraclass correlation coefficient of 0.90. We set the minimum acceptable intraclass correlation coefficient at 0.75. Based on these parameters and considering 2 repetitions per subject or 2 raters, we identified a minimum of 37 examinations to be included in the study, with a significance level of 0.05 and a power of 0.80.^16^, ^17^ We selected 40 examinations randomly from the database. The examinations were from patients with respiratory distress, patients without respiratory signs but with radiographic lung lesions (eg, lung metastasis), or animals admitted to the intensive care unit because of trauma or sepsis. The examinations were divided into 2 groups, “dogs” and “cats,” then anonymized and randomly selected. Forty examinations (20 for each group) were chosen following a randomization list, excluding those not correctly recorded.

All of the raters examined all of the anonymized videos independently, without coaching or consultation, assigning a score to each quadrant and determining a total score for every case. The raters repeated the score calculation on the same examinations after 3 months.

**FIGURE 1:**
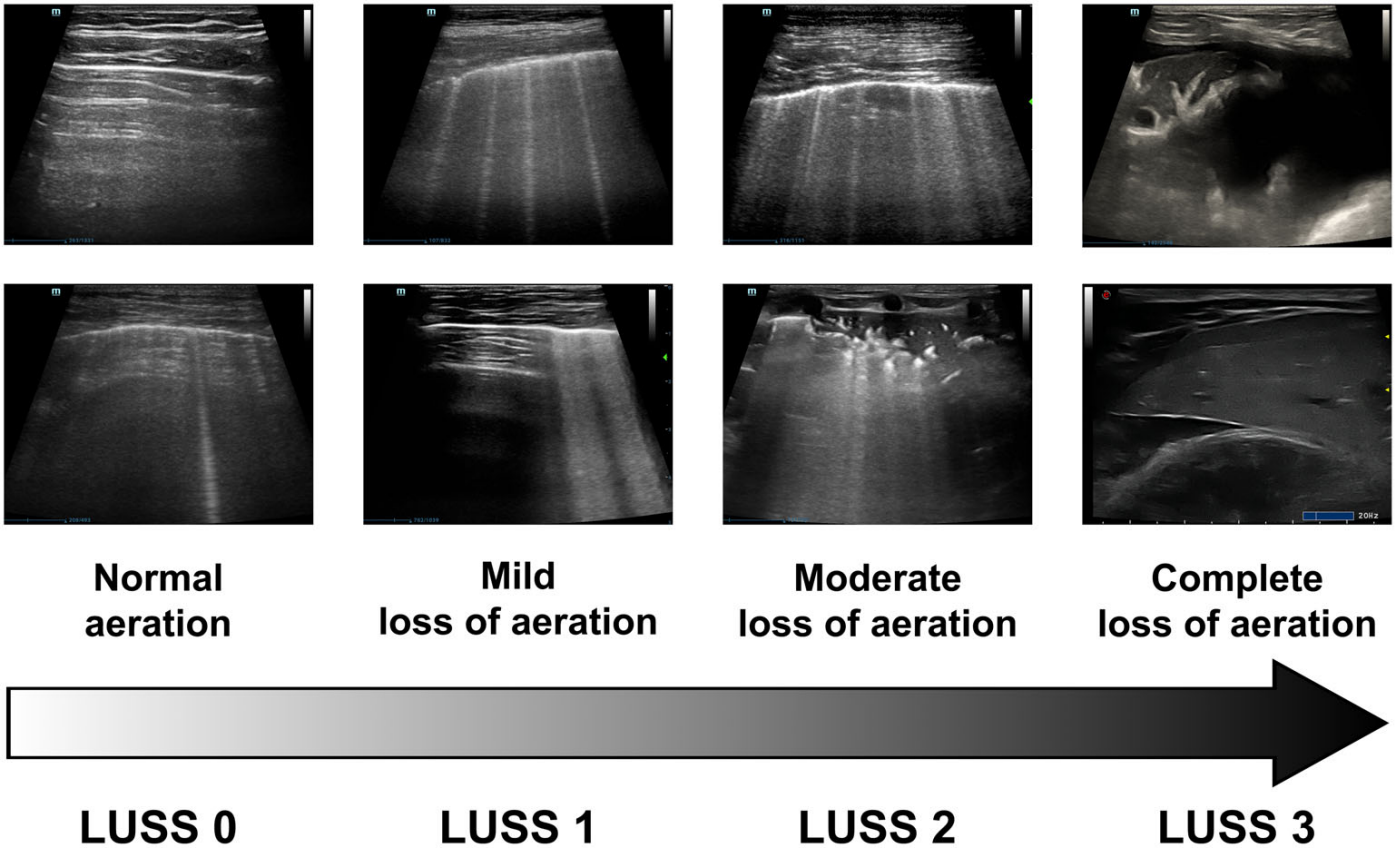
The image depicts the different levels of loss of aeration with LUSS, ranging from 0 (no loss) to 3 (complete loss due to atelectasis or consolidation). A score of 1 shows vertical artifacts covering 50% or less of the pleural surface, while a score of 2 shows vertical artifacts covering more than 50%. It is important to note that coalescent B‐Lines may not always be linked to score 2, as seen in LUSS 1.

According to a previous study,^4^ the score was assigned based on the percentage extension of the artifacts on the pleural surface, and each of the 8 quadrants was assigned a score ranging from 0 to 3 (Table 1, Figure 1):

LUSS = 0 (A‐profile) if there were horizontal artifacts (A‐lines) or a maximum of 2 vertical artifacts (ie, B‐lines).
LUSS = 1 if there were vertical artifacts (B1‐profile), or patterns such as pleural irregularity or slight subpleural loss of aeration that involved ≤50% of the visualized pleural line.
LUSS = 2 if there were vertical artifacts (B2‐profile) or subpleural loss of aeration or pleural effusion associated with loss of aeration (vertical artifacts or tissue‐like pattern) that involved >50% of the visualized pleural line.
LUSS = 3 if translobar tissue‐like pattern or pleural effusion occupied the entire visualized pleura without visualization of any aerated lung.

**TABLE 1:** Modality of the lung ultrasound score (LUSS) assignment.

LUSS 0	LUSS 1	LUSS 2	LUSS 3
100% of pleural surface occupied by	≤50% of pleural surface occupied by	>50% of pleural surface occupied by	≈100% of pleural surface occupied by
Horizontal artifacts (A‐lines) with ≤2 vertical artifacts (B‐lines)	Vertical artifacts (B1‐profile)	Vertical artifacts (B2‐profile) with or without pleural effusion (PE)	
	or	or	Translobar tissue‐like pattern (TrL‐TLP)
	Irregular pleural line (IPL)	Subpleural loss of aeration (SubPl‐LoA) with or without pleural effusion (PE)	or
	or		Pleural effusion (PE)
	Subpleural loss of aeration (SubPl‐LoA)		

The LUSS of 0 indicates normal aeration, whereas a score of 1 indicates mild loss of aeration; a score of 2 indicates moderate to severe loss of aeration, and a score of 3 indicates complete loss of aeration. To determine the total score, the scores of each quadrant were added together. Scores ranged from 0/24 for normal lungs to 24/24 for lungs with complete loss of aeration.

### 2.2 Pattern recognition agreement

Six months after the second rating (9 months after the beginning of the study), 40 individual videos were randomly selected from the 320 previously analyzed for further evaluation. The same 4 raters were tasked with identifying the prevalent pattern in the videos and describing every pattern they identified. To ensure accurate scoring, each observer had to record the presence (“yes”) or absence (“no”) of all of the listed patterns: A‐Lines (A), B‐Lines (B), irregular pleural line (IPL), subpleural loss of aeration (SubPl‐LoA), translobar tissue‐like pattern (TrL‐TLP), and pleural effusion (PE). By convention, because B1 is characterized by vertical artifacts that involve ≤50% of the visualized pleural line,^4^ in B1, the prevalent pattern was defined as A, whereas in B2, it was described as B. Moreover, each rater had to identify which of these artifacts or ultrasound signs represented the predominant one (“main pattern”) seen in the video, with all other patterns present (“yes”) being termed “secondary patterns.”

### 2.3 Statistical analysis

Statistical analyses were performed using commercially available software (MedCalc Version 22.002, Ostend, Belgium, IBM SPSS Statistics Version 29.0.1.0 [171] and Jamovi Version 2.3.28.0). Descriptive statistics were used for species, sex, age, and assigned scores. Data distribution was assessed both graphically and analytically. The Shapiro‐Wilk test was used to determine if the continuous variables were normally distributed. Based on their distribution, results were presented as mean ± SD or median and interquartile range (IQR; 25‐75th percentile). Intrarater and interrater reliabilities of the LUSS were assessed by intraclass correlation coefficient (ICC) and were presented with confidence interval (CI) as ICC (95% CI). For the intrarater ICC analysis, 2 measurements of each observer were used, and a single measurement, absolute agreement, and 2‐way mixed effects model was used. For the interrater ICC analysis, an average measurement, absolute agreement, and 2‐way random effects model was used to explore the reliability of 2 raters (each less experienced rater compared to H‐Exp) and 4 raters. The results were defined as poor if ICC was <0.5, moderate if ICC was 0.50 to 0.75, good if ICC was 0.75 to 0.90, and excellent if ICC was >0.90.^18^ A Spearman correlation between H‐Exp and the other raters was tested and plotted, and presented as rho (95% CI).

A Bland‐Altman plot and Spearman's rank correlation were used to explore the agreement and relationship between H‐Exp and the other 3 raters.

Cohen's kappa was utilized to assess the interrater reliability between 2 raters (H‐Exp and others) in identifying the prevalent pattern in each video, and considering the different patterns analyzed as nominal rather than ordinal variables, an unweighted kappa coefficient was chosen.^19^ Fleiss' kappa was used to assess the interrater reliability in identifying the prevalent pattern in each video among all of the raters. To be more conservative, kappa coefficients (k) were categorized as none (k: 0‐0.20), minimal (k: 0.21‐0.39), weak (k: 0.40‐0.59), moderate (k: 0.60‐0.79), strong (k: 0.80‐0.90), and almost perfect (k: >0.90) as proposed previously.^20^

Cochran's *Q* test was used to measure the raters' consistency in finding the same patterns (main and secondary) in every video.

Values of *P* < .05 were considered significant for all analyses.

## 3 RESULTS

The examiners reviewed 320 videos, with all 40 patients having 8 videos covering all 4 quadrants.

Of the 40 selected animals (20 dogs and 20 cats) the median age of dogs was 128 months (IQR, 83‐171 months), whereas cats had a median age of 83 months (IQR, 12‐153). Both groups had 8 females and 12 males. The dogs were a mixture of breeds, including 6 mongrels, 3 Chihuahuas, 3 Dachshunds, 2 Pinschers, 1 Epagneul Breton, 1 Golden Retriever, 1 Jack Russell terrier, 1 Labrador Retriever, 1 Poodle, and 1 Spitz. On the other hand, all of the cats were domestic short hair. The 40 examinations chosen at random included various conditions: cardiogenic pulmonary edema (10 dogs and 1 cat), pneumonia (4 dogs and 6 cats), pleural effusion (7 cats; 4 cardiogenic, 1 chylothorax, 1 hemothorax, and 1 pyothorax), neoplasia (3 cats), noncardiogenic pulmonary edema (2 dogs), pulmonary contusion (1 dog and 1 cat), pulmonary hypertension, pulmonary thromboembolism and pulmonary hemorrhage (1 dog each), lung abscess (1 cat), and multiple trauma with normal lung (1 cat).

**FIGURE 2:**
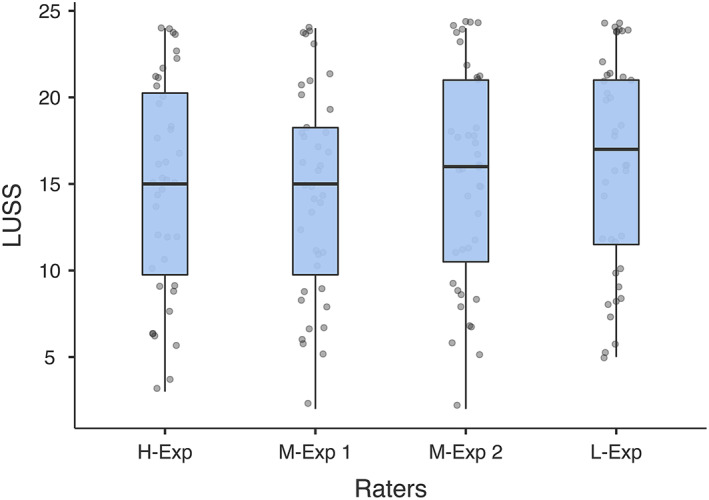
Box‐Plots showing all LUSS assigned to all cases by the four raters. In the plot, the points represent all the assigned scores for every rater, the boxes the interquartile range (25° lower and 75° upper), and the whiskers extend from each quartile to the minimum and the maximum. LUSS, lung ultrasound score.

The median score for the raters was 16 (IQR, 10‐21) for all of the examinations, 14 (IQR, 9‐16) for the dogs and 21 (IQR, 12‐24) for the cats (Figure 2).

The main and secondary patterns assigned by all of the raters are presented in Table 2.

**TABLE 2:** Main and secondary patterns assigned by the raters.

	Main patterns				Secondary patterns			
	H‐Exp	M‐Exp 1	M‐Exp 2	L‐Exp	H‐Exp	M‐Exp 1	M‐Exp 2	L‐Exp
A	10 (25.0%)	8 (20.0%)	9 (22.5%)	9 (22.5%)	12	17	12	13
B	13 (32.5%)	12 (30.0%)	16 (40.0%)	14 (35.0%)	16	16	13	16
IPL	2 (5.0%)	4 (10.0%)	1 (2.5%)	1 (2.5%)	18	12	18	19
SubPl‐LoA	3 (7.5%)	4 (10.0%)	2 (5.0%)	4 (10.0%)	7	12	11	7
TrL‐TLP	5 (12.5%)	5 (12.5%)	5 (12.5%)	5 (12.5%)	4	5	4	2
PE	7 (17.5%)	7 (17.5%)	7 (17.5%)	7 (17.5%)	6	4	5	2

*Note*: The main and secondary patterns assigned by each rater are presented in the table. There are a total of 40 main patterns (40 videos) that each rater needs to evaluate. These patterns are represented as numbers and percentages out of 40. On the other hand, all secondary patterns assigned by every rater to each video (which could be more than one per video) are expressed only as numbers because the total number of secondary patterns may vary between raters.

Abbreviations: A, A‐Lines; B, B‐Lines; IPL, irregular pleural line; PE, pleural effusion; SubPl‐LoA, subpleural loss of aeration; TrL‐TLP, translobar tissue‐like pattern.

The intrarater and interrater reliabilities were excellent, with an ICC >0.90 for all comparisons within and among observers. The intrarater ICC were 0.967 (CI 95%, 0.939‐0.982) for H‐Exp, 0.963 (CI 95%, 0.929‐0.981) for M‐Exp 1, 0.952 (CI 95%, 0.910‐0.975) for M‐Exp 2, and 0.950 (CI 95%, 0.896‐0.974) for L‐Exp. The reliability among all raters was excellent, with an ICC value of 0.980 (CI 95%, 0.966‐0.989). We compared the scores of 3 raters—2 with medium experience (M‐Exp 1 and M‐Exp 2) and 1 with low experience (L‐Exp) to the highly experienced operator (H‐Exp). The ICC values between H‐Exp and M‐Exp 1, M‐Exp‐2, and L‐Exp were 0.971 (CI 95%, 0.946‐0.985), 0.986 (CI 95%, 0.972‐0.992), and 0.955 (CI 95%, 0.866‐0.981), respectively. Intra‐ and interrater reliability was still very high when considering subanalyses of only dog or cat cases, with an ICC >0.90 for all analyses (Table S1).

**FIGURE 3:**
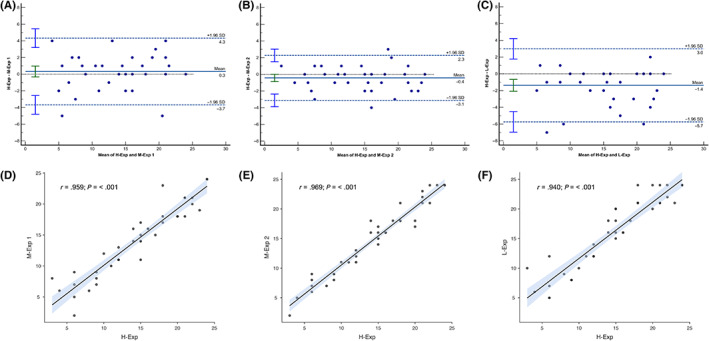
Bland‐Altman plots (above) of the LUSS assigned by the M‐Exp 1 (A), M‐Exp 2 (B), and L‐Exp (C), compared to the H‐Exp. The solid blue line represents the mean bias and the dashed blue lines indicate the 95% limits of agreement. The green and blue error bars display the 95% confidence intervals for the mean difference and the limits of agreement, respectively. Scatter plots (bottom) describing the correlation between M‐Exp 1 (D), M‐Exp 2 (E), and L‐Exp (F) LUSSs, compared to the H‐Exp LUSS.

The Bland‐Altman analysis showed a nonfixed bias (*P* = 0.32) of 0.325 (CI 95%, −0.328 to 0.978) with CI 95% limits of agreement from −3.679 and 4.329 between H‐Exp and M‐Exp 1, a nonfixed bias (*P* 0.06) of −0.425 (CI 95%, −0.865 to 0.0149) with CI 95%, limits of agreement from −3.121 and 2.271 between H‐Exp and M‐Exp 2 and a fixed bias (*P* < .001) of −1.375 (CI 95%, −2.087 to 0.663) with CI 95% limits of agreement from −5.739 and 2.989 between H‐Exp and L‐Exp (Table 3; Figure 3).

**TABLE 3:** Bland‐Altman analysis between H‐Exp and less experienced raters.

H‐Exp − M‐Exp 1		95% confidence interval		
	Estimate	Lower	Upper	
Bias (n = 40)	0.325	−0.328	0.978	*P* = .32
Lower limit of agreement	−3.679	−4.805	−2.553	
Upper limit of agreement	4.329	3.203	5.455	

H‐Exp − M‐Exp 2		95% confidence interval		
	Estimate	Lower	Upper	
Bias (n = 40)	−0.425	−0.865	0.015	*P* = .06
Lower limit of agreement	−3.121	−3.879	−2.363	
Upper limit of agreement	2.271	1.513	3.029	

H‐Exp − L‐Exp		95% confidence interval		
	Estimate	Lower	Upper	
Bias (n = 40)	−1.375	−2.087	−0.663	*P* < .001*
Lower limit of agreement	−5.739	−6.967	−4.512	
Upper limit of agreement	2.989	1.762	4.217	

*Note*: The Bland‐Altman method calculates the mean difference between two methods of measurement (the “bias”) and 95% limits of agreement as the mean difference (1.96 SD); the values represent the underestimating (positive value) and the overestimating (negative value) of the less experienced raters compare to the H‐Exp.

Abbreviations: H‐Exp, high‐experience rater; L‐Exp, low experience; M‐Exp, medium‐experience.

* Significant bias (*P* < .05).

The H‐Exp LUSS score was strongly and positively correlated (Figure 3) with M‐Exp 1 (rho, 0.959; *P* < .001), M‐Exp 2 (rho, 0.969; *P* < .001), and L‐Exp LUSS (rho, 0.940; *P* < .001).

The agreement between H‐Exp and the less experienced raters to find the main pattern was strong, with a Cohen's kappa 0.874 (CI 95%, 0.757‐0.991) between H‐Exp and M‐Exp 1, 0.869 (CI 95%, 0.747‐0.991) between H‐Exp and M‐Exp 2, and 0.839 (CI 95%, 0.707‐0.970) between H‐Exp and L‐Exp. The agreement among all the raters to identify the main pattern, as measured by Fleiss' kappa, was strong,^20^ with a kappa value of 0.855 (CI 95%, 0.791‐0.919; *P* < .001), considering all of the variables. Table 4 reports the agreements on the single variables (A, B, IPL, SubPl‐LoA, TrL‐TLP, PE).

**TABLE 4:** Agreements on the single variables between all the raters in identifying the prevalent pattern in each video.

Rating category	Fleiss' kappa	Asymptotic			Asymptotic 95% confidence interval	
		SE	*z*	*P*	Lower bound	Upper bound
A	.881	.065	13.641	**<.001**	.754	1.007
B	.806	.065	12.487	**<.001**	.680	.933
IPL	.474	.065	7.338	**<.001**	.347	.600
SubPl‐LoA	.693	.065	10.736	**<.001**	.566	.820
TrL‐TLP	1.000	.065	15.492	**<.001**	.873	1.127
PE	1.000	.065	15.492	**<.001**	.873	1.127

*Note*: Agreement significantly different from 0 (*P* < .05).

Abbreviations: A, A‐Lines; B, B‐Lines; IPL, irregular pleural line; PE, pleural effusion; SubPl‐LoA, subpleural loss of aeration; TrL‐TLP, translobar tissue‐like pattern.

Results of Cochran's *Q* test used to explore the consistency between raters to find all (main and secondary) patterns are reported in Table 5. The only significant finding was a significant difference in the detection of pleural effusion among raters, but only when comparing all of them. However, when excluding L‐Exp, Cochran's *Q* test showed nonsignificant results with a decreasing *Q* value from 8.08 (*P* = .04) to 3.00 (*P* = .22).

**TABLE 5:** Differences among all raters in identifying all patterns (main and secondary) in every video.

	Cochran's *Q*	*P*
A	5.40	.15
B	2.40	.49
IPL	3.69	.30
SubPl‐LoA	7.55	.06
Trl‐TLP	3.80	.28
PE	8.08	**.04** *

Abbreviations: A, A‐Lines; B, B‐Lines; IPL, irregular pleural line; PE, pleural effusion; SubPl‐LoA, subpleural loss of aeration; TrL‐TLP, translobar tissue‐like pattern.

* Significant differences (*P* < .05).

## 4 DISCUSSION

We evaluated the reliability of the LUSS in assessing lung aeration and the consistency of pattern recognition among raters with different levels of experience in LUS.

We found excellent reliability among all of the raters in the calculation of the LUSS as well as the identification of different patterns.

Lung ultrasound score had good reproducibility with excellent intrarater and interrater reliability, with an ICC >0.90. Our results confirm the easy applicability of this tool for the quantification of loss of aeration in dogs and cats in the emergency room and intensive care unit, not only in the diagnostic phase but also during patient monitoring. A dog with cardiogenic pulmonary edema should be monitored for several hours, evaluating lung decongestion during treatment, and LUSS may need to be performed by different operators. As well as excellent intrarater reliability, our data shows that, even between different raters, the scores are repeatable, which is crucial in a clinical setting. The results of the Bland‐Altman plot indicate a very low discrepancy between the H‐Exp and the raters with less experience, with a nonfixed bias for H‐Exp with M‐Exp‐1 and M‐Exp‐2 and a fixed bias for H‐Exp with L‐Exp, but with a value <2, considered a clinically acceptable score gap.^21^ Moreover, the rho value close to 1 indicates a very strong correlation among the LUSS of all of the raters compared to the H‐Exp's LUSS (Figure 3 and Table 3.

The agreement between H‐Exp and the raters with less experience in identifying the main pattern was strong, with a few exceptions. All of Cohen's kappa values were >0.8. The predominant patterns with almost perfect agreement among raters were pleural effusion and translobar tissue‐like pattern with kappa equal to 1 (Table 4), followed by A‐lines and B‐lines with strong agreement among raters (Table 4). The identification of subpleural loss of aeration showed a moderate level of agreement, whereas the irregularity of the pleural line had a weak level of agreement. The lower agreement in identifying normal pattern (A) and interstitial syndrome (B), compared to pleural effusion and translobar tissue‐like pattern, could be related to the challenge of distinguishing between B1 and B2 at times. Identifying the irregular pleural line artifact was most challenging, probably because of some raters' difficulty distinguishing between subpleural loss of aeration and irregularity of the pleural line when the former was small. Distinguishing between pleural irregularity and peripheral consolidation or atelectasis can be challenging, especially if the loss of aeration is not focal, well‐defined, or surrounded by homogeneous pleural artifacts. However, pleural irregularity generally appears thin and spread throughout the quadrant, whereas peripheral loss of aeration is more extensive in depth.^22^ In any event, the impact of this type of differentiation (small SubPl‐LoA vs irregularity of the pleural line) on the final LUSS is unlikely to be clinically relevant. Although all of the raters had excellent agreement in identifying pleural effusion as a prevalent pattern, the only significant difference observed was in identifying minimal effusions (secondary pattern), with a lower agreement for 1 rater (L‐Exp). However, these small pleural effusions were not clinically relevant.

In small animal veterinary medicine, many different scores have been described previously. Some studies have focused on counting B‐lines without examining the entire thoracic surface.^7^, ^8^, ^9^, ^10^, ^11^ Others referred to lung regions or quadrants or intercostal spaces, with a more extended analysis of the entire pleural surface.^12^, ^13^, ^15^ Only a few studies analyzed ultrasonographic signs other than B‐lines (eg, consolidations).^13^, ^14^ These scoring systems however were applied only to a particular disease or clinical scenario. In any event, all of these studies, regardless of the scanning techniques, were based on counting B‐lines, which could not differentiate the severity of the loss of aeration in the instance of a total number of B‐lines >3 and coalescent B‐lines. As a consequence, to better estimate extravascular lung water, it is suggested to evaluate the percentage of pleural line covered by B‐lines instead of counting the number of B‐lines on each ultrasound scan.^23^ Many in the medical field have adopted this type of LUSS,^4^, ^21^, ^24^, ^25^, ^26^, ^27^, ^28^ because of its ability to semiquantify aeration loss without overestimating it, avoiding counting of every B‐line in each quadrant. Furthermore, counting B‐lines is feasible when they are singular and isolated but becomes impractical beyond a certain threshold, even with software assistance,^29^ and this type of quantification could not be assigned to alveolar syndrome with a tissue‐like pattern. Additionally, in humans, when determining the amount of pleural surface involved (whether ≤50% or >50%), there is a stronger relationship with extravascular lung water (*R* ^2^ = 0.85) compared to the total number of B‐lines observed (*R* ^2^ = 0.0003).^23^ By dividing the thorax into quadrants, the technique allows for assessing the entire lung lobe rather than only specific points. Furthermore, it enables rapid scoring of the lung and pleural artifacts without counting individual artifacts. Standardizing the methodology when training is crucial, especially for scores 1 and 2. If the method for assigning the score is not clear to the rater (eg, how to define the threshold between B1 and B2 profiles), discrepancies may occur, which can be misleading in the scoring system, as shown before.^4^ This distinction is based on the extent of the pleural line affected by vertical artifacts or subpleural loss of aeration, not on the presence of coalescent B‐lines. Sometimes, even when there are only confined coalescent B‐lines or small subpleural loss of aeration, they are incorrectly classified as score 2 (B‐Lines) or 3 (SubPl‐LoA), leading to an overestimation of the overall score and the decreased lung aeration. Unquestionably, multiple coalescent B‐lines are linked to a more substantial loss of aeration than having only single B‐lines. However, if >50% of the pleural surface is affected by single B‐lines, it can lead to a more substantial loss of aeration compared to coalescent B‐lines that occupy <50% of the pleural surface.^4^ The pleural line is crucial in LUS scoring, and recently pleural line artifacts have been suggested by some authors as integrating the LUSS,^24^, ^30^, ^31^ and therefore attention should be paid to the pleural surface involved.

This scoring system allows rapid application of a semiquantification of lung aeration loss, with the possibility of being applied to all lung or pleural space pathology. When there is a complete loss of aeration in the entire explored quadrant, without seeing any portion of aerated lung, a score of 3 is to be given, regardless of whether it is caused by pleural effusion or a translobar lung tissue‐like pattern (consolidation or atelectasis). Conversely, if the signs only affect part of the pleural surface, a score of 1 or 2 depends on whether it is > or <50%.

In veterinary medical literature, no consensus exists regarding the best probe to use. One study^32^ described strong agreement between microconvex and phased‐array to analyze B‐lines, whereas another study^8^ showed greater agreement for the linear and microconvex probes, compared to a phased‐array probe, with an interchangeable use for linear and microconvex probes when different patterns (pulmonary edema, pneumonia, lung neoplasia) were analyzed. For several years in our center, LUS has been performed using a linear probe to analyze more pleural surface which helps prevent pleural blurring, false thickening of the bright white line, and any increase in the lateral size of vertical artifacts. Vertical artifacts are more common with the convex probe, which, because of a fan‐shaped mode, makes the ultrasound beam not orthogonal to the pleura.^33^ Furthermore, LUSS is based on the analysis of the pleural surface and therefore, the linear probe has high frequency and high superficial resolution and allows for better exploration without altering the shape of the pleura line, unlike the convex probe. How the probe is positioned is an important factor affecting the measured score. One study^4^ found that changing the probe from a longitudinal to a transverse scan resulted in a decrease in the number of regions rated with a score of 0 and an increase in the number rated with a score of 2. This result likely occurred because a transversal scan, which runs parallel to the ribs' long axis, allows for better exploration of the pleural surface compared to a longitudinal scan that encounters rib barriers. Operators must follow the same procedure during examinations to ensure consistency in pattern recognition and prevent variations in the results of the LUSS.

In clinical practice, we chose a 4‐quadrant scheme for the scoring system, unlike the 6‐area system used in humans,^4^ because of the different topographic anatomy and different thorax morphology between humans and dogs or cats. In dogs and cats, the edges between ventral, lateral, and dorsal are not so clear as in humans (anterior, lateral, and posterior), based on anterior and posterior axillary lines. Furthermore, based on our experience, subdividing the thorax into many small areas, especially in cats or small dogs, can lead to scanning the same place twice with a linear probe, altering the final value of the LUSS and overestimating or underestimating it depending on the type of ultrasound sign present.

Our study had some limitations. To test reliability, we used archived videos recorded by a single expert operator, but we did not test the interrater variability associated with image acquisition and scan performance. Moreover, it was important for us to include pleural effusion in the scoring system because it can cause moderate to severe atelectasis, particularly in cats, which affects the aeration of the lungs. However, doing so may create some confusion between the pathophysiology of intra‐ and extralung diseases, and as a result, limit the applicability of LUSS as a diagnostic tool for certain diseases. Diagnosis should be made by pattern recognition rather than LUSS, which is meant to evaluate the severity of aeration loss and for monitoring purposes. The context of the pathophysiology of the respiratory distress and the chronicity of the disease must be considered as well, because patients with chronic disease may have more severe loss of aeration with fewer clinical signs compared to those with acute disease. Another limitation could be the exclusion of some examinations not correctly recorded (too short videos with rapid scans, videos in which the contact between the probe and the skin was lost, examinations without videos). This factor might undermine interrater reliability in high‐stress clinical scenarios, where patients are difficult to restrain and scan. In any event, these factors could have an impact on LUSS as a whole, not just the reliability among raters. Additionally, in point‐of‐care ultrasound,^34^ it is crucial to combine the clinical examination, medical history, and ultrasound results for accurate diagnosis. Therefore, interpreting ultrasound images in the context of clinical signs and findings can be helpful in guiding diagnosis. This information was not provided to the raters, who viewed the multimedia material without any information apart from the animal species. Finally, there was no time limitation for viewing videos, unlike an emergency situation. Furthermore, as part of the validation process, in addition to the intrarater and interrater reliability, a test of agreement with respiratory function tests and advanced diagnostic imaging could be helpful.

In conclusion, our results indicate excellent intra‐ and interrater reliability for LUSS and pattern identification, providing a foundation for the use of LUS in emergency medicine and intensive care.

## CONFLICT OF INTEREST DECLARATION

Authors declare no conflict of interest.

## OFF‐LABEL ANTIMICROBIAL DECLARATION

Authors declare no off‐label use of antimicrobials.

## INSTITUTIONAL ANIMAL CARE AND USE COMMITTEE (IACUC) OR OTHER APPROVAL DECLARATION

Authors declare no IACUC or other approval was needed.

## HUMAN ETHICS APPROVAL DECLARATION

Authors declare that human ethics approval was unnecessary for this study.

## Supporting information


**Table S1.** Intrarater and interrater reliabilities are expressed with the intraclass correlation coefficient (CI 95%). ICC values were presented for all the population and the two subgroups of dogs and cats. ICC: intraclass correlation coefficient (CI 95%).
