# Supplementary material for: Lung ultrasound score in dogs and cats: A reliability study
Source: J Vet Intern Med. 2023 Nov 27;38(1):336–45. doi: 10.1111/jvim.16956 (PMC10800220; doi:10.1111/jvim.16956)
Supplement: Supplementary file 1 — Table S1. Intrarater and interrater reliabilities are expressed with the intraclass correlation coefficient (CI 95%). ICC values were presented for all the population and the two subgroups of dogs and cats. ICC: intraclass correlation coefficient (CI 95%). [file JVIM-38-336-s001.pdf]

Supplementary Table 1

Intra-raters

Raters	All	Dogs	Cats
H-Exp	.967 (CI 95% .939 - .982)	.968 (CI 95% .921 -.987)	.960 (CI 95% .904 - .984)
M-Exp 1	.963 (CI 95% .929 - .981)	.947 (CI 95% .655 -.984)	.965 (CI 95% .914 - .986)
M-Exp 2	.952 (CI 95% .910 - .975)	.912 (CI 95% .792 -.964)	.957 (CI 95% .868 - .984)
L-Exp	.950 (CI 95% .896 - .974)	.922 (CI 95% .809 -.968)	.957 (CI 95% .887 - .983)

Inter-raters

Raters	All	Dogs	Cats
All	.980 (CI 95% .966 - .989)	.981 (CI 95% .961 - .991)	.976 (CI 95% .948 - .990)
H-Exp - M-Exp 1	.971 (CI 95% .946 - .985)	.975 (CI 95% .936 - .990)	.966 (CI 95% .914 - .986)
H-Exp - M-Exp 2	.986 (CI 95% .972 - .992)	.982 (CI 95% .956 - .993)	.984 (CI 95% .958 - .994)
H-Exp - L-Exp	.955 (CI 95% .866 - .981)	.959 (CI 95% .863 - .985)	.941 (CI 95% .782 - .979)

Intra-rater and inter-rater reliabilities are expressed with the intraclass correlation coefficient (CI 95%). ICC values were presented for all the population and the two subgroups of dogs and cats.

ICC: intraclass correlation coefficient (CI 95%)
